# Assessment of taste profile dynamics during crabapple (*Malus prunifolia* (Willd.) Borkh.) ripening by metabolomics and electronic tongue analysis

**DOI:** 10.3389/fnut.2026.1823361

**Published:** 2026-04-30

**Authors:** Xiao-Hua Dai, Jing Chen, Xiang-Ying Wei, Lu-Xia Ran, Feng-Jin Zheng, Usman Rasheed, Gan-Lin Chen

**Affiliations:** 1 Guangxi Subtropical Crops Research Institute, Guangxi Academy of Agricultural Sciences, Nanning, Guangxi, China; 2 Key Laboratory of Quality and Safety Control for Subtropical Fruit and Vegetable, Ministry of Agriculture and Rural Affairs, Nanning, Guangxi, China; 3 Guangxi Key Laboratory of Quality and Safety Control for Subtropical Fruits, Nanning, Guangxi, China; 4 Institute of Agro-Products Processing Science and Technology, Guangxi Academy of Agricultural Sciences, Nanning, Guangxi, China

**Keywords:** correlation analysis, electronic tongue, flavor, *Malus prunifolia* (Willd.) Borkh, metabolomics

## Abstract

Crabapple (*Malus prunifolia* (Willd.) Borkh) is valued for its ornamental appearance, effectiveness as a rootstock, and rich nutritional profile. However, the flavor profile and metabolite composition are poorly characterized, which hinders a comprehensive assessment of its overall value. This study combined broad-spectrum untargeted metabolomics with electronic tongue analysis to systematically characterize the flavor and metabolite profiles of *M. prunifolia* across four distinct ripening stages, thereby clarifying the relationship between flavor and metabolites and uncovering the metabolic basis of flavor formation. The results indicated that the fundamental flavor characteristics remained relatively stable throughout ripening, with an overall trend toward increased sweetness and reduced sourness, bitterness, and astringency. Among these attributes, sweetness and sourness were the primary indicators of ripeness. The increase in sweetness was associated with changes in fructose, glucose, sucrose, D-mannitol, and D-sorbitol, and the reduction in sourness was associated with changes in ketoglutaric acid, aconitate, as well as significant enrichment of glyoxylate and dicarboxylate metabolism. Diverse flavonoids (including quercitrin, astragalin, rutin, mangiferin, neohesperidin, catechin, and (−)-epicatechin), triterpenoids (including maslinic acid, corosolic acid, and alphitolic acid), and phenolic acids (including cryptochlorogenic acid) were identified. While these metabolites contribute to bitterness and astringency, and also impart substantial functional properties. The results provide a reference for assessing the nutritional value of *M. prunifolia*, facilitating resource utilization and targeted extraction of bioactive compounds.

## 1 Introduction

*Malus prunifolia* (Willd.) Borkh, also known as the crabapple, is a member of the genus *Malus* in the family Rosaceae (1). It is distributed across Asia, Europe, and North America and is widely cultivated in Xinjiang, China. This species is valued as an ornamental plant and as an excellent rootstock for grafting cultivated apples and other plants. Owing to its distinctive flavor and rich nutritional content, it has been developed into various food products such as wine, juice, vinegar, and syrup (2). Recent studies have investigated the composition, concentrations, and bioactive profiles of metabolites, demonstrating that *M. prunifolia* is rich in functional compounds, including chlorogenic acid, gallic acid, rutin, hyperoside, quercetin, and kaempferol (3–5). These compounds contribute to its notable medicinal value, strong antioxidant activity, and inhibitory effects on cancer cell proliferation (6). These studies provide a theoretical basis for developing new *M. prunifolia* resources, expanding its nutritional applications, and promoting it as a functional food. However, comprehensive analyses of its overall metabolite profile remain limited, and functional compounds beyond phenolics have been insufficiently investigated. This gap impedes a multidimensional assessment of its nutritional value and constrains progress in targeted breeding and novel product development.

The taste quality of fruits significantly influences their market value, and sensory evaluation is commonly used as a standard assessment method. Electronic tongue (E-tongue) technology converts electrical signals into data matrices to obtain taste information, enabling more objective, accurate, and rapid analysis of flavor quality (7). This technology has been widely applied in Rosaceae fruit research, primarily to distinguish varietal differences based on core flavor attributes (8–10), monitor taste changes during the later postharvest stages, assess postharvest flavor alterations, and establish correlations between flavor profiles and physicochemical indicators (11, 12). Currently, integrated evaluation approaches combining E-tongue, electronic nose, metabolomics, and transcriptomics have become significant areas of research interest. These combined analyses can identify metabolites that influence fruit flavor from multiple perspectives, clarify how changes in metabolite types and levels drive taste evolution, and establish a more comprehensive flavor quality evaluation system (10, 13, 14). Although *M. prunifolia* has been developed into a variety of food products, the flavor of its fresh fruit has not yet been quantified or objectively assessed. Moreover, studies exploring the relationship between its flavor profile and intrinsic metabolites remain scarce, limiting the enrichment of its evaluation framework and understanding of the chemical basis underlying its taste.

Ripening is a critical stage in fruit growth and development, with significant implications for quality, harvest timing, storage, transportation, and marketability. Differences in ripening stages involve diverse physiological changes, including variations in the types and concentrations of flavor compounds and functional metabolites (15). These metabolic changes can ultimately shape flavor during fruit ripening by regulating core taste attributes (15–17). These mechanisms have been extensively studied in Rosaceae and other fruits, including persimmon. During ripening, dynamic changes in soluble sugars, organic acids, and tannins collectively determine the intensity, character, and balance of fruit flavor (18–21). In a study related to *M. prunifolia*, Tu et al. (22) compared changes in moisture content, soluble solids, vitamin C, and other nutritional components across four *M. prunifolia* cultivars at different ripening stages, providing insights into nutrient variation trends during ripening as well as reference points for determining optimal harvest timing and consumption methods. However, this study did not examine the association between metabolite dynamics and taste attributes. Studies linking *M. prunifolia* ripening stages to functional metabolite profiles are scarce, and robust evidence supporting nutritional evaluation and optimal harvest time remains lacking.

In summary, this study combined broad-spectrum untargeted metabolomics with electronic tongue analysis to systematically characterize the metabolite and flavor profiles of fruits across four distinct ripening stages. By establishing correlations between taste profiles and physicochemical indicators, it clarifies how changes in metabolite composition and abundance drive flavor evolution, thereby providing a theoretical basis for nutritional evaluation, graded resource utilization, and targeted extraction of bioactive compounds.

## 2 Materials and methods

### 2.1 Plant materials

**Figure 1:**
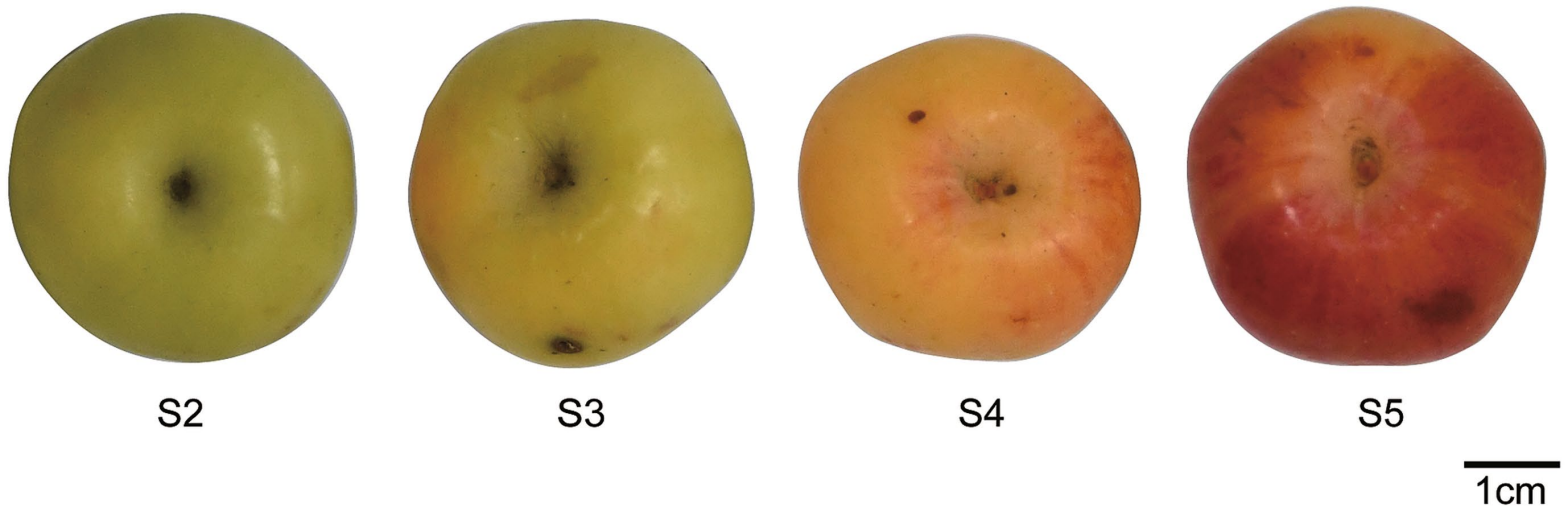
Representative samples of crabapple fruits at four maturity stages.

*M. prunifolia* (crabapple) fruits were collected from Xinjiang Agricultural University in August 2024. The region has a temperate continental climate with low rainfall, abundant sunshine, and large diurnal temperature variation. Fruits were selected according to a previously established protocol (23). Briefly, fruits were categorized as follows: predominantly yellow-green, red pigmentation covering 1/4th of the total fruit surface, yellowish where red pigmentation covers ½ of the fruit surface, and fully ripened red fruits. Ten healthy, independently fruiting trees were randomly selected. From the upper to middle canopy of each tree, 5–10 fresh, uniformly sized, undamaged fruits were collected. The fruits were preliminarily classified into four maturity stages: S2 (coloration covering ≤1/4 of the total fruit surface, predominantly yellow green), S3 (coloration covering ≥1/2 of the total surface, predominantly yellow), S4 (full surface coloration, predominantly yellow with partial red blush), and S5 (fully colored, entirely red) (Figure 1). Each maturity stage included three biological replicates (*n* = 3) and five technical replicates to ensure adequate statistical power. The samples were rinsed with ultrapure water and 70% ethanol to remove surface contaminants and then dried with gentle air. A portion of the sample was deseeded and cut into small pieces. The remaining whole fruits were flash-frozen in liquid nitrogen and stored at −80 °C for further analysis.

### 2.2 Analysis of metabolites and metabolomics data processing

Sample preparation, extract analysis, metabolite identification, and quantification were conducted by VeryGenome Technology Co., Ltd. (Guangzhou, China) in accordance with their standardized protocols and established methodologies. Briefly, fruits were homogenized using a lab grinder, and precisely 100 mg of each sample was accurately weighed and mixed with 1 mL of extraction solvent (methanol: acetonitrile: water = 2:2:1, v/v). The reaction mixture was subjected to ultrasonic extraction in an ice-water bath for 10 min, followed by flash-freezing in liquid nitrogen for 1 min. This process was repeated three times. The samples were then stored at −20 °C for 1 h, followed by centrifugation. The supernatant was collected, dried under a gentle stream of nitrogen gas, and reconstituted in 100 μL of acetonitrile: water (1:1, v/v). The reconstituted samples were further sonicated in an ice-water bath for 15 min, then centrifuged, and the supernatant was collected for mass spectrometry analysis.

An ultra-high-performance liquid chromatography-mass spectrometry (UPLC-MS) system, equipped with a Waters UPLC BEH Amide column (2.1 × 100 mm, 1.7 μm), was used for liquid chromatography separations and mass spectrometry detection. An injection volume of 5 μL was utilized under specific conditions, with the column temperature set to 55 °C. The mobile phase gradient included water with 25 mM ammonium acetate and 25 mM ammonium hydroxide (designated as phase A) along with 100% acetonitrile (designated as phase B). The gradient was programmed as follows: from 0 to 1 min, 85% B; from 1 to 12 min, 65% B; from 12 to 12.1 min, 40% B; from 12.1 to 15 min, 40% B; from 15 to 15.1 min, 85% B; and finally, from 15.1 to 20 min, 85% B. The flow rate was consistently maintained at 0.3 mL/min. Mass spectrometry data acquisition conditions were as follows: electrospray ionization (ESI) with an ion source voltage of 4,500 V or 5,500 V; curtain gas (CUR) at 20 psi; and nebulizing and auxiliary gases at 60 psi each.

Raw UPLC-MS data were converted to mzXML format using ProteoWizard, followed by peak alignment, retention time correction, and peak area analysis with the SCIEX OS software package. Metabolite identification was performed using both primary and secondary spectral matching (within 25 ppm), referencing the proprietary VeryGenome database as well as public metabolomic databases. Metabolite quantification was performed using Multiple Reaction Monitoring (MRM) mode, with peak area integration utilized for standardizing the data.

Pairwise comparisons of metabolites were conducted across the four different maturity stages of *M. prunifolia*, designated as S2 vs. S3, S3 vs. S4, and S4 vs. S5. Metabolic features were initially analyzed using orthogonal partial least squares discriminant analysis (OPLS-DA) and hierarchical clustering analysis (HCA). Differentially accumulated metabolites (DAMs) were identified using the following criteria: a variable importance in projection (VIP) score of ≥1, an absolute fold change of >1.2 or <0.8, and a *p*-value of <0.05. The identified metabolites were then annotated with information from the Kyoto Encyclopedia of Genes and Genomes (KEGG) database to explore their functional pathways. Statistical analysis and visualization were conducted using R software using ggplot, pheatmap and plotly packages.

### 2.3 Electric tongue taste evaluation

Taste analysis was performed using the SA-402B electronic tongue system (INSENT, Japan). Fresh fruit samples weighing 10 grams were homogenized in 400 milliliters of deionized water and then centrifuged at 1,000 RPM for 5 min. A total of 40 milliliters of the supernatant was collected for instrumental analysis. The E-tongue system was employed to assess five primary taste attributes: sweetness, sourness, bitterness, astringency, umami, and saltiness, alongside aftertaste signals such as aftertaste-A, aftertaste-B, and richness. For each sample, four replicates were used for primary taste attributes. Five replicates were performed for sweetness measurement, with the average of the last three stable responses used as the final taste signal. Taste evaluation was conducted by comparing taste values to the established threshold levels. The E-tongue thresholds were calibrated using the instrument’s standard solutions and represent the critical points for taste detection, ensuring analysis accuracy: −13 for sourness, −6 for saltiness, and 0 for all other tastes. A taste value exceeding its threshold indicated the presence of that taste, with higher values reflecting greater intensity (7, 24). Preliminary data analysis was conducted using principal component analysis (PCA), OPLS-DA, and radar plots to identify differences among the samples. Statistical analyses were conducted in R, with a one-way analysis of variance (ANOVA) used to evaluate significant differences among groups. Tukey’s test was used for *post-hoc* comparisons, with *p* < 0.05 considered statistically significant.

## 3 Results

### 3.1 Flavor analysis of *Malus prunifolia*

**Figure 2:**
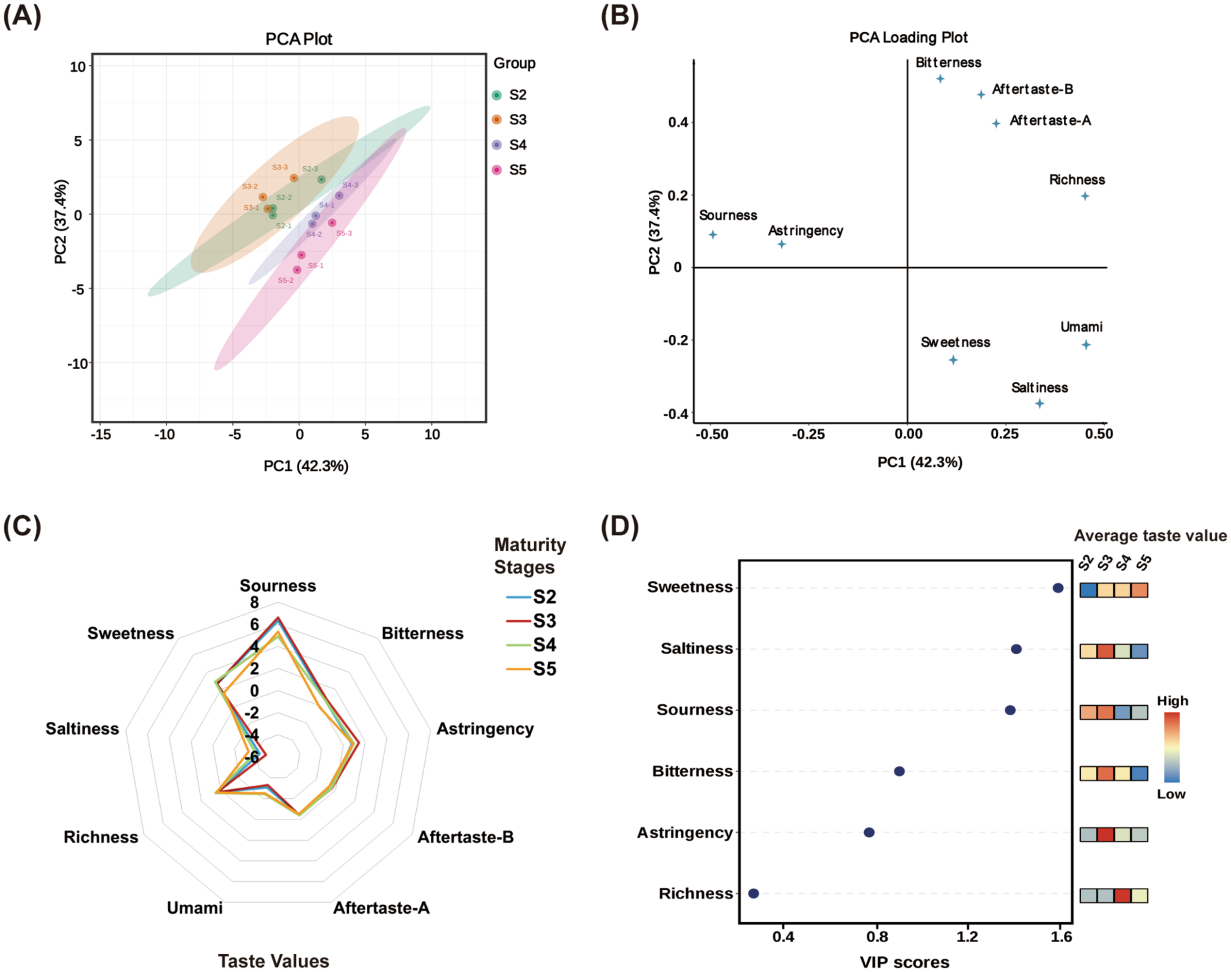
Multidimensional analysis of taste characteristics in crabapple fruits across four maturity stages. PCA score plot based on the taste profiles **(A)**; the PCA loading plot **(B)**; taste radar chart **(C)**; variable importance in projection (VIP) from PLS-DA, visualized for taste indicators with meaningful taste values only **(D)**.

The taste profiles of *M. prunifolia* fruits at different ripening stages were evaluated using an E-tongue, providing an objective physicochemical basis for sensory characterization (7, 25). As shown in Figure 2C, the taste radar plots and response intensities were similar across ripening stages. The aftertaste of bitterness (aftertaste-B), aftertaste of astringency (aftertaste-A), and umami values were below 0, indicating that these taste attributes were not relevant for characterization. In stages S2–S4, sourness, bitterness, umami, saltiness, sweetness, and richness values were all above the tastelessness threshold, indicating measurable taste signals. In stage S5, all taste values except bitterness were above the threshold. Sourness exhibited the highest taste value, indicating that it is the dominant taste attribute in ripe *M. prunifolia* fruits. As shown in Figures 2A,B, the samples displayed good within-group clustering but no distinct separation between groups, suggesting that overall taste profiles were relatively similar across the four ripening stages. To further determine the contribution of each detectable taste attribute, importance prediction was conducted. As shown in Figure 2D, sweetness and sourness had VIP values greater than 1, identifying them as the primary taste components distinguishing the four ripening stages. Comparative analysis of taste responses revealed that sweetness increased progressively with ripening, while sourness, bitterness, and astringency showed a declining trend.

### 3.2 Comprehensive metabolomic analysis

**Figure 3:**
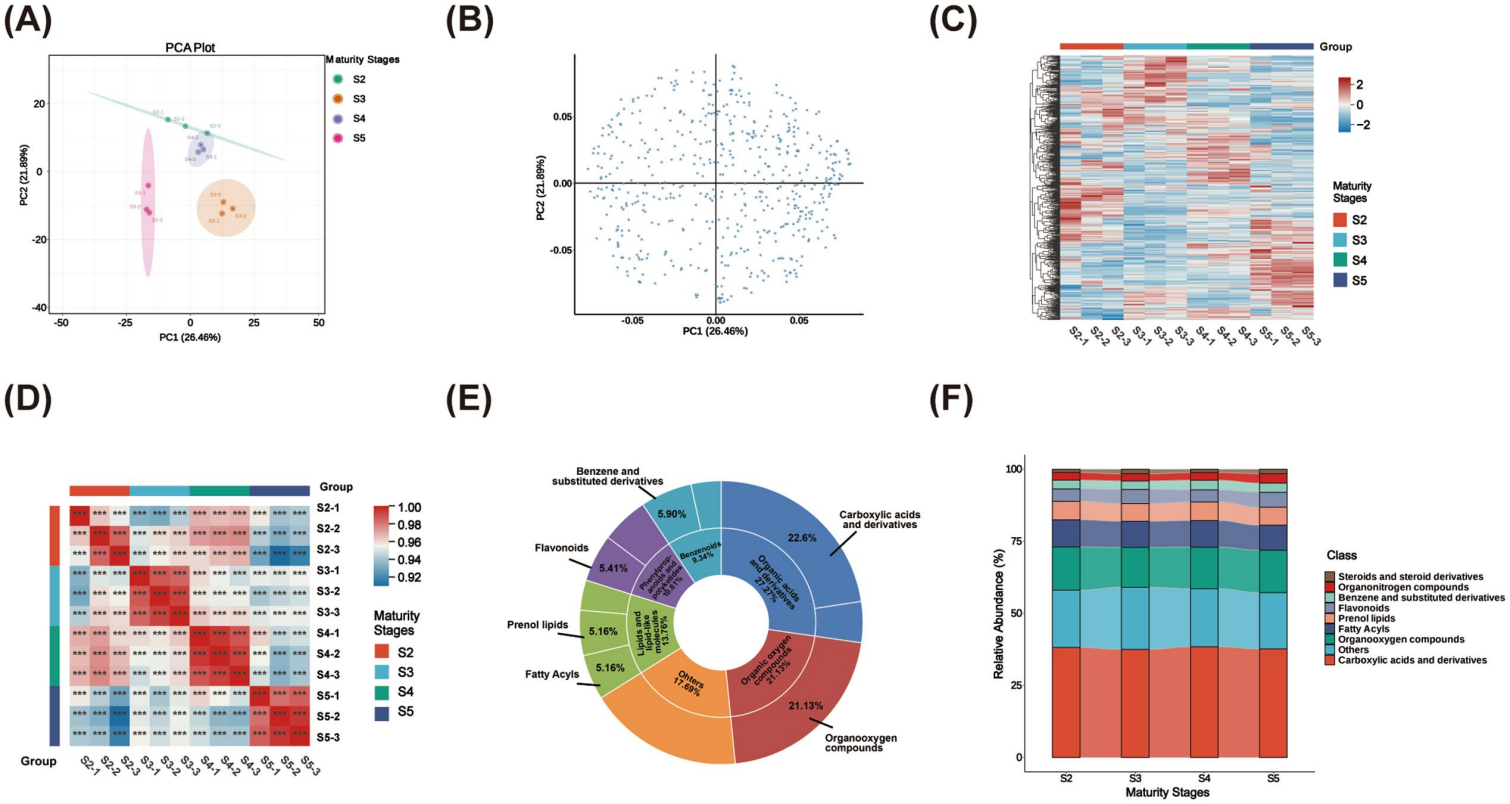
Multivariate statistical analysis in crabapple fruits across four maturity stages. PCA score plot based on relative metabolite abundance **(A)**; the PCA loading plot **(B)**; hierarchical cluster analysis (HCA) of metabolite content across groups **(C)**; Intragroup correlation analysis **(D)**; pie chart of identified metabolites. The outer ring represents “Super class”; categories with a proportion lower than 9.34% are grouped into “Others.” The inner ring represents “Class,” with only those >5.16% annotated **(E)**; stacked bar chart with connecting lines **(F)**.

To elucidate the metabolite composition of ripe *M. prunifolia* fruits, UPLC-MS was used to profile metabolites at four stages of ripening. As shown in Figures 3A,B, samples displayed good within-group clustering and a clear separation trend among groups (PC1: 26.46%, PC2: 21.89%). The metabolomic profiles of fruits at different ripening stages exhibited distinct differences (Figure 3C). Data reliability analysis indicated correlation coefficients greater than 0.8, with all annotated thresholds being highly significant (*p* < 0.001), confirming that the metabolomics dataset was reproducible and suitable for further analysis (Figure 3D). A total of 407 metabolites were identified, primarily including 92 carboxylic acids and derivatives (22.60%), 86 organooxygen compounds (21.13%), 24 benzene and substituted derivatives (5.90%), 22 flavonoids (5.41%), 21 fatty acyls (5.16%), and 21 prenol lipids (5.16%) (Figure 3E and Supplementary Table S1). A stacked bar chart was used to illustrate the relative proportions and variation trends of the main metabolite classes. The levels of carboxylic acids, organooxygen compounds, and benzene derivatives remained consistent throughout ripening (Figure 3F), indicating that these essential flavor characteristics were largely unaffected by ripening.

### 3.3 Differential metabolomic analysis

**Figure 4:**
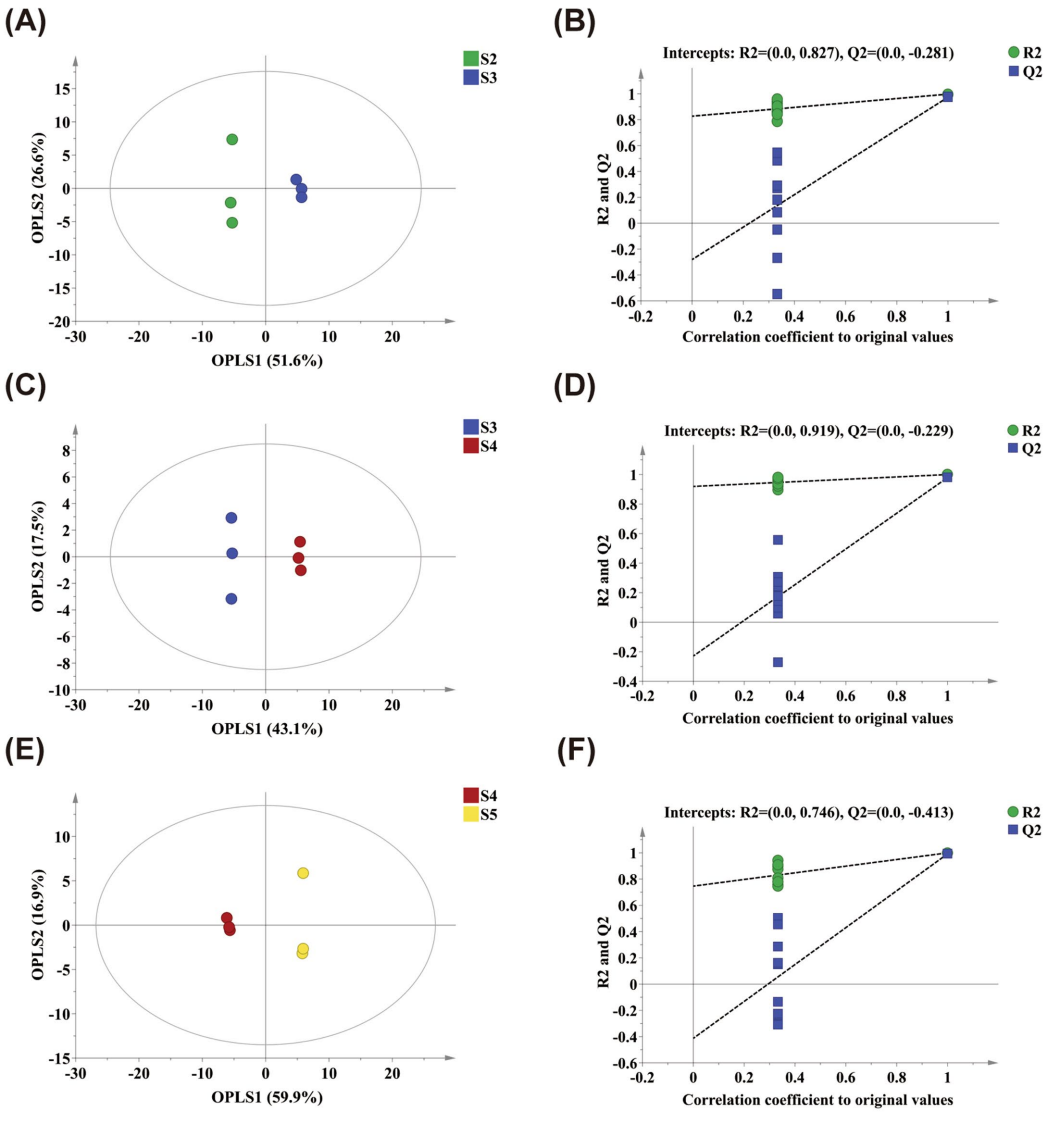
Multivariate statistical analysis of differentially accumulated metabolites (DAMs). OPLS-DA score plots **(A,C,E)**; permutation test of the OPLS-DA models **(B,D,F)**. The comparisons are as follows: S2 vs. S3 **(A,B)**, S3 vs. S4 **(C,D)**, S4 vs. S5 **(E,F)**.

To further characterize metabolic differences among *M. prunifolia* fruits at various ripening stages, OPLS-DA analysis was performed. Clear separation was observed for all stage comparisons (S2–S5), which indicates significant differences in metabolic profiles across different stages of maturity (Figures 4A,C,E). Model stability and reliability were further validated through 200 permutation tests. The results showed negative *Q*^2^*Y* intercept values, with the original models having higher *R*^2^*Y* and *Q*^2^*Y* values than the corresponding permutation models, and no evidence of overfitting. These findings confirm the suitability of the models for subsequent screening of differential metabolites (Figures 4B,D,F).

**Figure 5:**
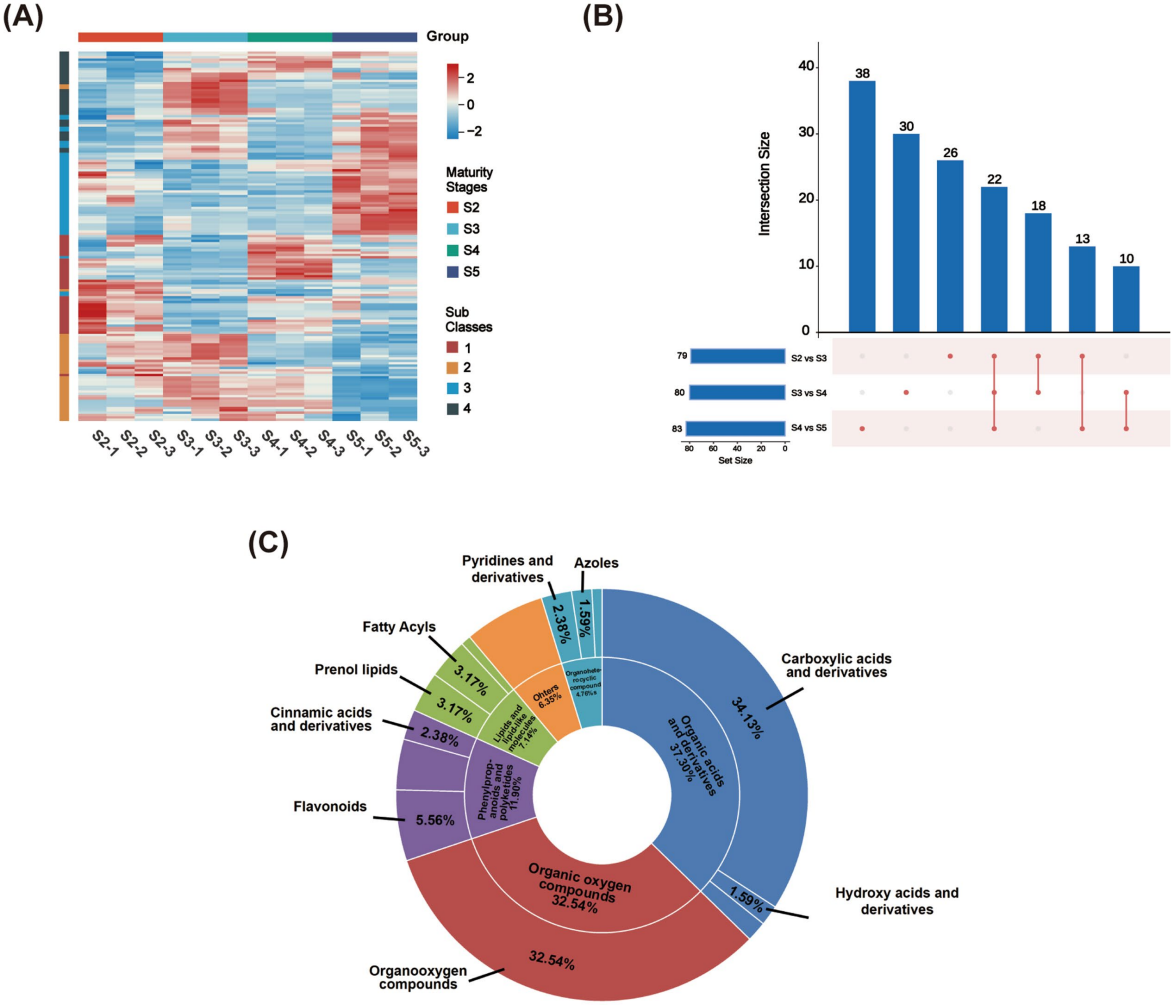
Multivariate statistical analysis of DAMs. Hierarchical cluster analysis (HCA) of DAMs content across groups **(A)**; the UpSet chart of DAMs **(B)**; pie chart of identified metabolites. The outer ring represents “Super class”; categories with a proportion lower than 4.76% are grouped into “Others.” The inner ring represents “Class,” with only those >1.59% annotated **(C)**.

To analyze metabolite accumulation patterns across the four ripening stages, we screened for differential metabolites (DAMs) using the following criteria: VIP >1, *p* < 0.05, and a fold change (FC) greater than 1.2 or less than 0.83. We identified a total of 157 DAMs, which primarily included carboxylic acids and their derivatives (34.13%), organooxygen compounds (32.54%), and flavonoids (5.56%) (Figure 5C and Supplementary Table S2). Co-expression correlation analysis was performed to elucidate intrinsic relationships among DAMs (Supplementary Table S3). As shown in Figure 5A, DAMs displayed distinct accumulation patterns across ripening stages. Comparisons of S2 vs. S3, S3 vs. S4, and S4 vs. S5 yielded 79, 80, and 83 DAMs, respectively, with 26, 30, and 38 unique DAMs in each comparison, and 22 DAMs shared across all comparisons (Figure 5B). Using differential intensity quantitative analysis, importance was assessed by the mean VIP of DAMs across stages, with values of 1.8789 (S2 vs. S3), 1.8832 (S3 vs. S4), and 1.8427 (S4 vs. S5). Intensity was evaluated by the mean |log_2_(FC)|, which was 0.5354, 0.5635, and 0.5025, respectively. Core high DAMs were further screened using VIP >2, *p* < 0.05, and FC >2 or <0.5, and their proportions among DAMs were 24.05, 30.00, and 26.51%, respectively. These results indicate that the largest metabolic changes occurred at S3 vs. S4 (Supplementary Table S2).

**Figure 6:**
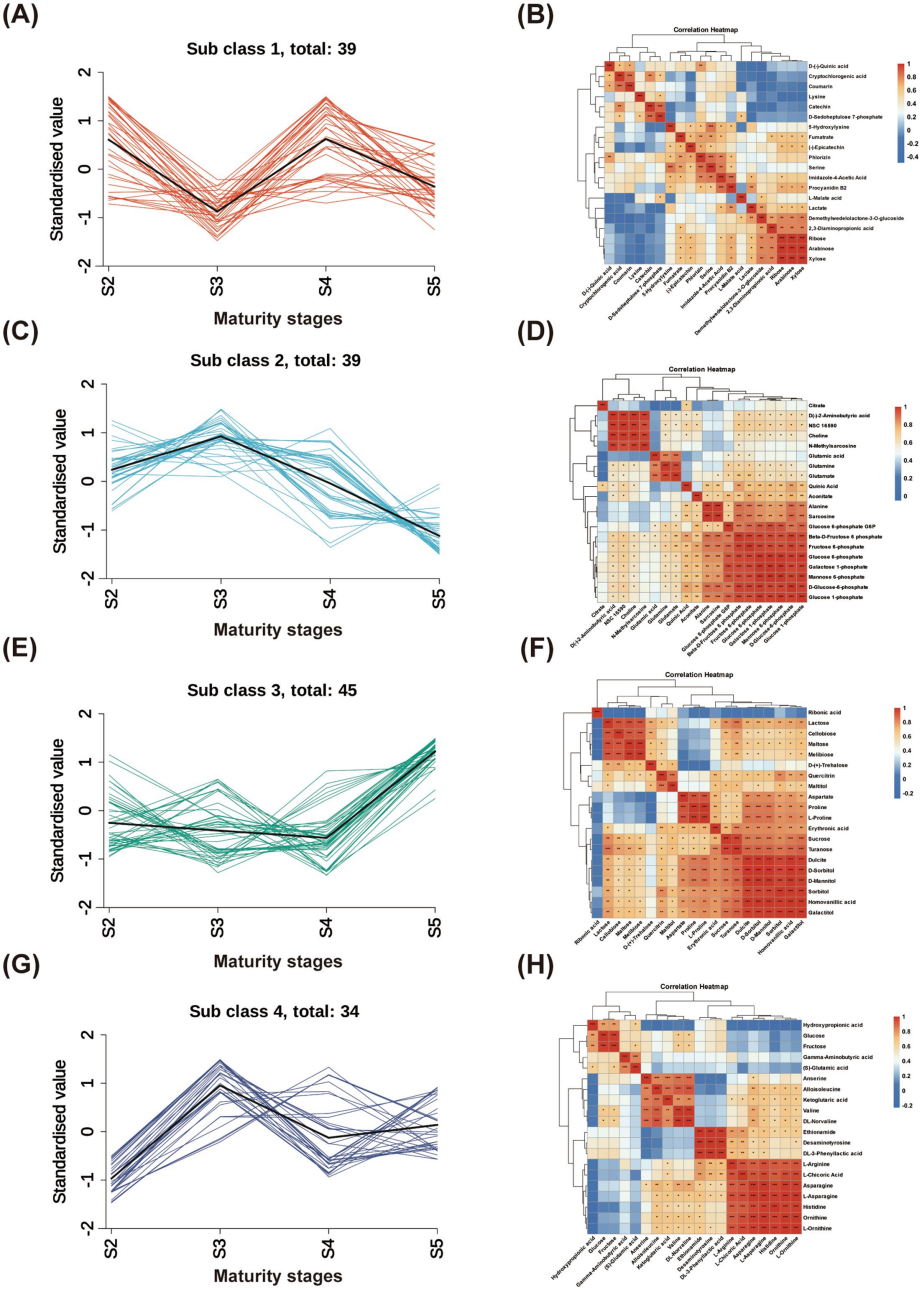
K-means cluster analysis of DAMs **(A,C,E,G)** and correlation analysis of each cluster **(B,D,F,H)**. Pearson’s correlation coefficient was used to calculate correlations, and the top 20 were selected for visualization. Statistical significance is indicated as follows: ^*^p < 0.05, ^**^p < 0.01, and ^***^p < 0.001.

K-means clustering was applied to the DAMs to group metabolites with similar variation patterns into four clusters, followed by correlation analysis within each cluster. As shown in Figure 6 and Supplementary Table S2, metabolites exhibiting an increasing trend with ripening were primarily concentrated in subclass 3. This group included sucrose, turanose, dulcitol, D-mannitol, D-sorbitol, and sorbitol, which were significantly correlated with each other and showed marked accumulation at stage S5. Metabolites that began to decline from stage S3 were mainly clustered in classes 2 and 4, including quinic acid, aconitic acid, and ketoglutaric acid. Compounds in class 1 exhibited a decrease followed by an increase, including catechin, (−)-epicatechin, cryptochlorogenic acid, and procyanidin B2, with several displaying significant correlations within the group (Supplementary Table S2).

**Figure 7:**
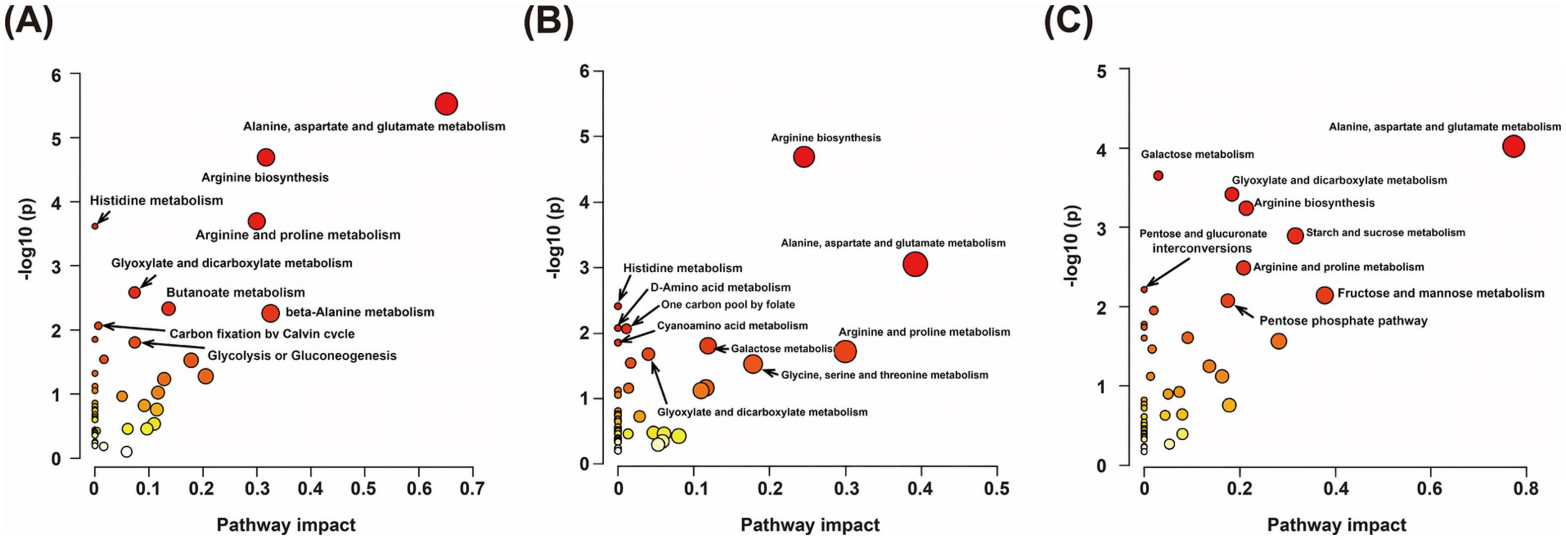
The bubble plot chart shows the KEGG enrichment results of DAMs in three comparison groups: S2 vs. S3 **(A)**, S3 vs. S4 **(B)**, and S4 vs. S5 **(C)**. Bubble size represents the number of differential metabolites enriched in the corresponding pathway, and the color indicates the significance level (−log_10_ (*p*)), with darker colors representing more significant enrichment.

To further elucidate the global characteristics and regulatory hubs of *M. prunifolia* metabolites, discrete DAMs were mapped onto system-level biological function networks. The KEGG enrichment results for the comparisons S2 vs. S3, S3 vs. S4, and S4 vs. S5, as shown in Figures 7A–C, revealed several overlapping metabolic pathways. These pathways include the metabolism of alanine, aspartate, and glutamate; glyoxylate and dicarboxylate metabolism; arginine biosynthesis; arginine and proline metabolism; cyanoamino acid metabolism; and nicotinate and nicotinamide metabolism. Notably, in S4 vs. S5, significant enrichment was observed in key carbohydrate-related pathways such as starch and sucrose metabolism and fructose and mannose metabolism, which are central to sugar composition and accumulation (19).

### 3.4 Integrated analysis of electronic tongue and differential metabolites

To elucidate the relationships between taste attributes and metabolites in *M. prunifolia*, DAMs were correlated with sweetness, sourness, bitterness, and astringency values through co-expression correlation analysis (Supplementary Tables S3, S4).

Sweetness was significantly associated with sucrose, glucose, fructose, turanose, D-sorbitol, D-mannitol, and dulcitol (*r* > 0.62, *p* < 0.05). Notably, glucose and fructose levels increased significantly from stage S3, while sucrose, turanose, D-sorbitol, D-mannitol, and dulcitol showed marked increases at stage S5. These results were consistent with the accumulation patterns observed in subclass 3, and most of these compounds were significantly correlated within groups (*p* < 0.05). This indicates that the compounds associated with sweetness collectively underlie the increase in sweetness during *M. prunifolia* ripening.

Bitterness was positively correlated with maslinic acid, alphitolic acid, and corosolic acid (*r* > 0.65, *p* < 0.05), but negatively correlated with quercitrin, astragalin, rutin, catalpol, mangiferin, neohesperidin, and arbutin (*r* < 0.64, *p* < 0.05). Although these metabolites were not among the top 20 in the correlation ranking for cluster 1, intragroup correlation analysis revealed strong associations among several flavonoids: quercitrin with astragalin and rutin (*r* > 0.92, *p* < 0.001), and astragalin with rutin (*r* = 0.93, *p* < 0.001). This suggests that the persistent bitterness observed during *M. prunifolia* ripening may result from dynamic changes in these compounds.

Sourness showed negative correlations with 4-hydroxybenzoic acid, urocanic acid, salicylic acid, and shikimic acid (*r* < 0.63, *p* < 0.01). Intragroup analysis revealed significant correlations among these metabolites: salicylic acid with 4-hydroxybenzoic acid and urocanic acid (*r* > 0.83, *p* < 0.001), urocanic acid with 4-hydroxybenzoic acid (*r* = 0.71, *p* < 0.01), and shikimic acid with salicylic acid (*r* = 0.64, *p* < 0.05). Based on the preceding results, this suggests that tartness development in *M. prunifolia* depends not only on organic acid accumulation but also on the coordinated involvement of multiple metabolic pathways.

**Figure 8:**
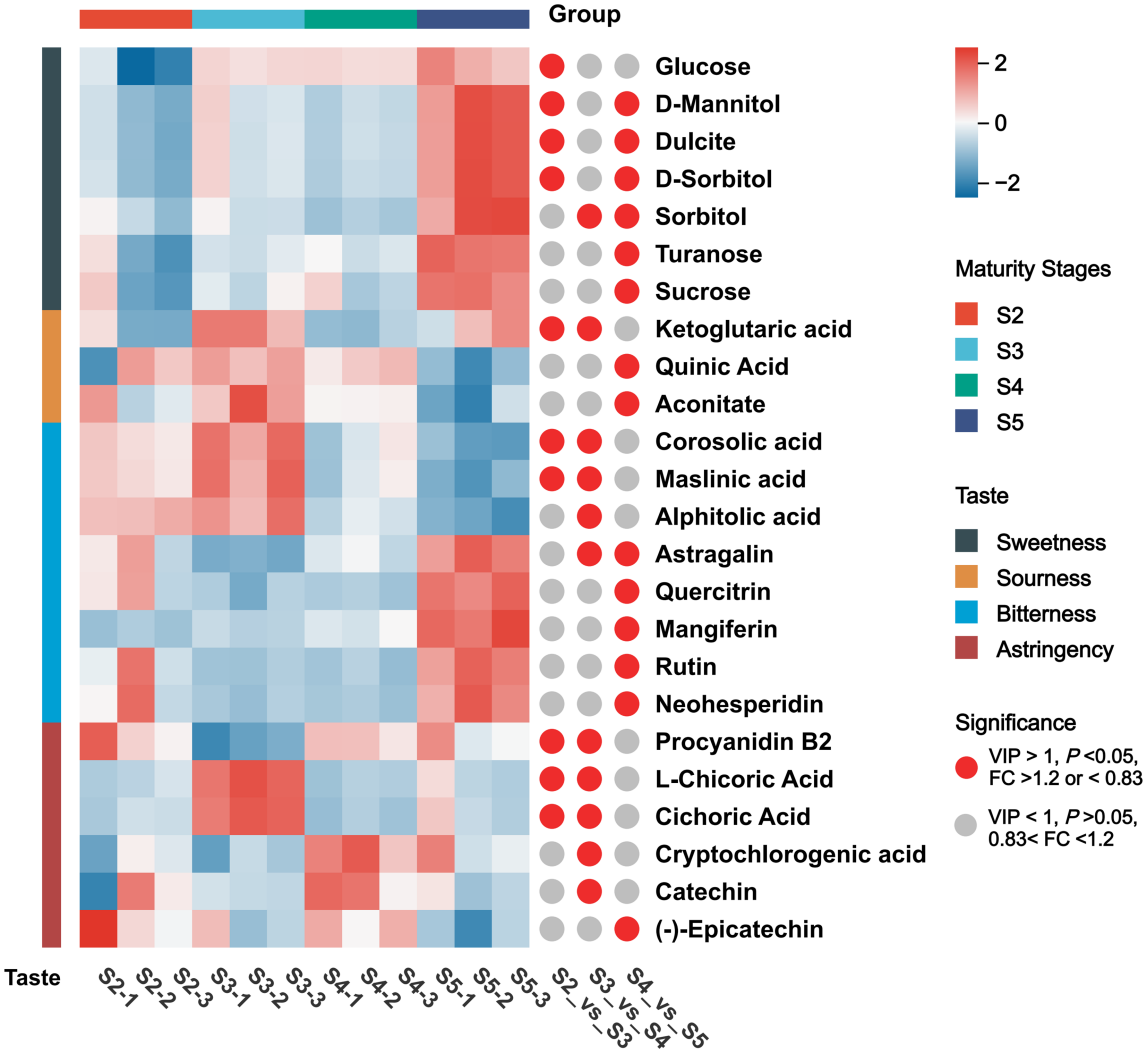
Hierarchical cluster analysis (HCA) of key metabolite concentrations.

Astringency was positively correlated with L-chicoric acid and cichoric acid (*r* > 0.61, *p* < 0.05). These two compounds showed a very high correlation (*r* = 0.99, *p* < 0.001). This indicates that the development and variation of astringency in *M. prunifolia* result from the synergistic regulation of multiple phenolic acids and their derivatives. Finally, Figure 8 provides a clear, intuitive illustration of the compounds significantly associated with flavor, along with dynamic changes in their relative concentrations across different ripening stages.

## 4 Discussion

### 4.1 Overall metabolite and taste profile of *Malus prunifolia*

Taste, aroma, color, and nutritional composition are key determinants of fruit quality. The sweet–sour balance, primarily governed by sugars and organic acids, is a critical factor influencing flavor perception (26–30).

In the present study, broad-spectrum untargeted metabolomics was employed to profile metabolites in *M. prunifolia* fruits at different ripening stages. A variety of flavor-related compounds were identified, including organic acids and their derivatives, organic oxygen compounds, lipids, and lipid-like molecules. The relative abundances of these compounds remained largely stable across ripening stages, indicating that the fundamental flavor components of *M. prunifolia* are rich yet relatively unchanged during maturation. From a postharvest handling and processing perspective, *M. prunifolia* fruits allow flexible selection of maturity stage to match specific processing needs. Core flavor attributes remain stable over short periods, supporting consistent respiratory metabolism and reducing flavor deterioration. Stable lipid and lipoid contents help maintain membrane integrity, delaying senescence and moisture loss. These findings provide scientific support for optimizing harvest timing and extending shelf life (31).

Furthermore, we used an E-tongue to evaluate the taste profiles of *M. prunifolia* fruits at different ripening stages, providing an objective physicochemical basis for sensory characterization (7, 25). Comparative analysis of taste response values identified sourness as the dominant taste attribute in ripe fruits. This finding aligns well with the metabolomic results, which revealed abundant and stable levels of organic acids, thereby confirming from both sensory and analytical perspectives that organic acids constitute the core chemical basis of sourness in *M. prunifolia*. Beyond directly determining fruit acidity, organic acids also shape flavor complexity, palatability, and processing quality (32, 33). In processing applications, they can maintain low pH to enhance effective antimicrobial action, prevent non-enzymatic browning, act as fermentation agents, or serve as curing agents (32).

We also found that sweetness is a key flavor attribute of ripe *M. prunifolia* fruits. Together with the many oxygen-containing organic compounds identified by metabolomics, this provides a potential chemical basis for the formation of sweetness. Sweetness is not isolated; the balance between sweetness and sourness largely determines overall flavor. Moderate sweetness enhances palatability and offsets the sharpness of organic acids, resulting in a more harmonious taste profile, which aligns with the common processing strategy of adjusting the sweetness-to-sourness ratio (26, 27, 34).

However, flavor formation is a complex process that involves gene regulation, metabolite accumulation, and multi-component interactions. Assessing only taste and metabolite profiles at ripening cannot fully explain the mechanisms and regulatory pathways of flavor development. Therefore, analyzing dynamic metabolite changes together with molecular regulation can address the limitations of studies focused on static traits. For example, Zhang et al. (30) used HPLC-MS to track sugars and organic acids during apple development and found that most sugars accumulated at rates faster than or comparable to fruit growth, whereas organic acids accumulated more slowly. Similarly, Yang et al. (29) reported coordinated changes in sugars, acids, and flavonoids, along with their associated gene expression patterns, during cherry ripening. Therefore, our results not only provide a theoretical reference for understanding the flavor characteristics of *M. prunifolia* but also establish a scientific basis for future flavor improvement and processing applications.

### 4.2 Integrated analysis of differential metabolites and sensory attributes in *Malus prunifolia*

Across ripening stages, the metabolites in *M. prunifolia* primarily comprised carboxylic acids and derivatives, organooxygen compounds, and flavonoids, which largely reflected the overall metabolite profile. These metabolites have been widely reported to be closely associated with fruit flavor. Carboxylic acids and their derivatives, as key organic acid precursors, can directly modulate sourness through concentration changes (33). Some organooxygen compounds and their derivatives may contribute to sweetness accumulation (35), whereas certain flavonoids are often associated with bitterness and astringency (36, 37). We integrated quantitative indicators of change magnitude and significance and found that S3–S4 was the key transition stage. As reported for other fruits, the pre-maturity stage showed the largest metabolic differences, with bidirectional regulation of metabolites, indicating metabolic network restructuring (38, 39). This provides a temporal reference for elucidating metabolic regulation during the ripening of *M. prunifolia*. In terms of taste characteristics, sweetness and sourness were the most important and predictive factors for distinguishing the four ripening stages. Sweetness increased progressively with maturation, whereas sourness, bitterness, and astringency declined. Further analysis of the relationships between metabolites and taste attributes revealed that changes in core taste perceptions were closely aligned with dynamic shifts in metabolite levels, indicating that fluctuations in metabolite abundance drive changes in these sensory attributes (15–17). These findings provide a direct basis for more in-depth identification of key metabolites associated with sweetness, sourness, bitterness, and astringency.

#### 4.2.1 Relationship between differential metabolites and sweetness

Sweetness is the defining flavor attribute distinguishing the different ripening stages of *M. prunifolia*. The continuous accumulation of sugars during ripening directly drives the increase in sweetness and provides the fundamental basis for improved flavor quality and ripening-related variation. These metabolites included sucrose, glucose, fructose, turanose, D-sorbitol, D-mannitol, and dulcitol. Fructose is among the sweetest naturally occurring sugars, surpassing sucrose in sweetness, as it efficiently binds to sweet taste receptors and produces an intense sweet perception; it is also widely used in the food industry as a sweetness enhancer (40). Glucose, although common, has a lower sweetness intensity than fructose (41). From S2 to S3, both fructose and glucose contents increased significantly. Between S3 and S5, their levels remained high with only slight, non-significant increases. Previous studies have demonstrated that fructose and glucose are the predominant sugars in *M. prunifolia* fruits (35). In addition, sucrose, turanose, and mild-tasting sugar alcohols such as D-sorbitol and D-mannitol showed significant increases during the later ripening stages (41). KEGG enrichment analysis revealed that starch and sucrose metabolism as well as fructose and mannose metabolism pathways were significantly enriched. These pathways regulate sugar accumulation by degrading starch into soluble sugars, such as glucose and fructose, and by mediating the metabolism of fructose and mannose. Together, these processes determine the intensity, type, and overall balance of fruit sweetness (19).

The increase in sweetness during *M. prunifolia* ripening is the result of the combined action of multiple sweet-tasting compounds. The early accumulation of fructose and glucose establishes the sweetness foundation, which is then maintained and slightly enhanced in later stages through continued accumulation of sugar alcohols such as sorbitol and mannitol, collectively shaping the fruit’s overall sweetness profile. Similar to the sugar accumulation patterns observed in most Rosaceae fruits during ripening, sucrose, fructose, and glucose in apples, pears, and peaches accumulate coordinately and directly promote sweetness development, while sorbitol, in addition to functioning as a major transport sugar alcohol, also contributes directly to sweetness (19, 42, 43).

#### 4.2.2 Association between differential metabolites and sourness

Sourness is the dominant taste attribute of *M. prunifolia* fruits and a key flavor characteristic distinguishing the different ripening stages. As ripening progressed, overall acidity steadily declined, accompanied by similar decreases in ketoglutaric acid (2-oxoglutaric acid), aconitate (aconitic acid), and quinic acid (quinate), along with significant enrichment of the glyoxylate and dicarboxylate metabolism pathway. This pathway is a key regulator of organic acid accumulation in fruits, determining final acidity by coordinating the synthesis, storage, and degradation of organic acids (44). In this pathway, ketoglutaric acid participates in this pathway. Its content increased significantly during both S2–S3 and S4–S5, and its accumulation directly influences the synthesis rates of downstream succinic acid, malic acid, and citric acid, thereby contributing to the pronounced acidic profile and flavor complexity of *M. prunifolia* (45). Aconitate is also involved in this pathway as a key intermediate in the conversion of citrate to isocitrate in the TCA cycle, and its concentration is directly regulated by aconitase activity (44). In this study, elevated aconitate levels were observed from S2 to S4 and then declined sharply at S5, consistent with the decrease in sourness during fruit maturation. Although aconitate levels dropped, elevated ketoglutaric acid content continued to drive downstream organic acid synthesis, sustaining notable acidity in late-stage fruits. Additionally, we observed that quinic acid exhibits a dynamic profile similar to that of sourness. This metabolite has a strong sour taste and, at certain concentrations, contributes more to acidity than citric or malic acid (46). Similarly, high quinic acid levels in kiwifruit and blueberry contribute to their pronounced tart flavor profiles (46, 47). Peaches also contain substantial amounts of quinic acid and smaller amounts of aconitate, and these organic acids can influence tartness in a genotype-dependent manner (48).

Therefore, the contrasting trends of aconitate and quinic acid accumulation may partly explain the differences in sourness across ripening stages in *M. prunifolia*. These results indicate that fruit sourness is determined not by the absolute level of a single organic acid, but by the synergistic effects and dynamic balance of multiple organic acids within the metabolic network.

#### 4.2.3 The relationship between differential metabolites and bitterness

Bitterness is a persistent taste attribute throughout *M. prunifolia* ripening. Its composition changes dynamically, with triterpenoids contributing predominantly at the early stages and flavonoids becoming dominant at the later stages; together, these compounds sustain the bitter profile throughout ripening. We detected triterpenoids such as maslinic acid, corosolic acid, and alphitolic acid, which are secondary metabolites widely found in the plant and have been extensively studied and identified as one of the primary sources of bitterness. For example, triterpenoids are directly responsible for the bitterness of bitter melon (*Momordica charantia*), and this bitterness is associated with its traditional heat-clearing and detoxifying properties (49). Likewise, the triterpenoid saponin mixture abundant in the roots of red beet (*Beta vulgaris* L.) is the primary contributor to its intense bitterness (50). It is worth noting that triterpenoids also possess functional properties that effectively prevent and treat infectious diseases, chronic diseases, and cancer (51). Therefore, the decline in their abundance during ripening may partly explain the reduced bitterness of *M. prunifolia* fruits and may also be accompanied by changes in certain biological activities.

In addition, a small portion of the flavonoid detected in *M. prunifolia* maintained stable abundance throughout ripening. In the later stages, compounds such as quercitrin, astragalin, rutin, mangiferin, and neohesperidin were identified. Flavonoids are known for their strong antioxidant capacity and a broad range of health-promoting and pharmacological activities, including anti-inflammatory, anticancer, antiviral, and immunomodulatory effects. Due to these functional properties, they are widely applied in the food, cosmetics, and pharmaceutical industries (52–56). Yu et al. reported that the total flavonoid content in 12 *M. prunifolia* cultivars ranged from 20 to 100 mg/100 g RE, with antioxidant activity approximately 10 times higher than that of common fruits, and identified rutin as the predominant flavonoid (35). However, certain flavonoids impart pronounced bitterness that can influence flavor perception. For instance, flavonoid glycosides such as neohesperidin, rutin, quercetin, and naringenin have been shown to be key determinants of bitterness in citrus fruits (36, 37).

E-tongue analysis indicated that while ripe *M. prunifolia* fruits exhibit detectable bitterness, it is not a dominant taste attribute. The presence of abundant flavonoids and triterpenoids contributes only mildly to bitterness; their greater significance lies in their functional properties. In traditional Chinese medicine, bitterness is often associated with heat-clearing and detoxifying effects (57). The *Encyclopedia of Traditional Chinese Medicine* (ETCM) compiled documents 403 medicinal herbs, of which 240 are classified as bitter (58). Therefore, reducing bitterness while retaining functional components and improving other sensory attributes has become a key objective in fruit breeding and processing. Previous studies have employed approaches such as fungal fermentation and heterologous expression to obtain enzymes that remove bitter flavonoids while enhancing functionality and flavor. Therefore, reducing bitterness while retaining functional components and improving other sensory attributes has become a key objective in fruit breeding and processing. Previous studies have used approaches such as fungal fermentation and heterologous expression to obtain enzymes that remove bitter flavonoids while enhancing functionality and flavor (59–61). These technologies have been applied in commercial fruit juice production, achieving flavonoid reductions, such as for naringin, of 70% or more (62, 63). These findings provide preliminary data and practical strategies for targeted breeding of *M. prunifolia* and for developing efficient debittering approaches in processing industries.

#### 4.2.4 Correlation of differential metabolites with astringency

Astringency is a characteristic flavor attribute of *M. prunifolia* during ripening and is primarily determined by the dynamic levels of phenolic acids and their derivatives, as well as flavans, making it a key factor affecting fruit texture and flavor quality. We detected L-chicoric acid and cichoric acid, while the contents of cryptochlorogenic acid, catechin, (−)-epicatechin, and procyanidin B2 declined with ripening. These compounds are recognized as major contributors to plant astringency, producing a dry, puckering mouthfeel through interactions with proteins (64, 65). Previous studies have shown that catechin, (−)-epicatechin, and chlorogenic acid are representative phenolic compounds in *M. prunifolia* and play a major role in enhancing its antioxidant activity (4). Compared with apples, *M. prunifolia* contains higher levels of phenolic acids, including gallic, protocatechuic, chlorogenic, *p*-coumaric, and ferulic acids, as well as flavonoids such as quercetin and myricetin, thereby conferring significantly greater antioxidant activity (4, 5, 35). In other model systems, catechin is a key determinant of tea bitterness and astringency, with intensity increasing linearly with catechin concentration (66). Persimmons contain abundant procyanidins (condensed tannins), which are polymers of flavan-3-ol monomers such as catechin and epicatechin. Tannin content peaks during fruit enlargement and decreases sharply as fruits enter the ripening stage (20).

While excessive astringency reduces consumer acceptability, many astringency-associated compounds possess functional properties such as antioxidant activity, cardiovascular protection, and prevention of chronic diseases (5). To improve flavor while maintaining health benefits, many studies have used physicochemical treatments, regulation of biosynthetic pathways, molecular-targeted modulation, microbial treatments, and enzymatic processing to reduce or modify soluble tannins and other phenolics. This reduces their binding to oral proteins and achieves deastringency. These approaches are widely used in commercial persimmon production, reducing tannins by up to 90% and shortening debittering to 24–48 h while preserving phenolic bioactivity and bioavailability (65, 67). These approaches provide preliminary data and potential strategies for targeted breeding of *M. prunifolia* and for implementing efficient deastringency processes in the processing industry.

## 5 Conclusion

This study is the first to comprehensively characterize dynamic changes in flavor-related metabolites during *M. prunifolia* maturation using broad-spectrum untargeted metabolomics and E-tongue analysis. The results established a comprehensive metabolic landscape and revealed relatively stable dynamic patterns of fundamental flavor characteristics during maturation. They also established the relationships between the four primary and distinctive flavor attributes, sweetness, sourness, bitterness, and astringency, and their corresponding metabolites. Additionally, bioactive compounds contributing to bitterness and astringency were identified. These findings provide valuable references for nutritional evaluation, hierarchical resource utilization, precision processing, and targeted extraction of bioactive compounds in *M. prunifolia*. However, this study also has some limitations. It focused on a single regional variety, which may limit the broader applicability of the findings. In addition, relative quantitative data provide valuable insights into metabolite–flavor associations, although they do not fully establish direct contributions to flavor. Furthermore, the underlying biosynthetic and regulatory mechanisms would benefit from further investigation. In addition, future research can be directed toward practical applications, such as developing quantitative models linking metabolite content with taste indices, identifying and characterizing key genes regulating flavor traits, and optimizing targeted debittering and deastringency processing strategies. Such efforts will ultimately enhance the comprehensive utilization efficiency and industrial value of *M. prunifolia*.

## Data Availability

The original contributions presented in the study are included in the article/Supplementary material, further inquiries can be directed to the corresponding authors.
